# Sensitive Periods for Developing a Robust Trait of Appetitive Aggression

**DOI:** 10.3389/fpsyt.2015.00144

**Published:** 2015-10-13

**Authors:** Anke Köbach, Thomas Elbert

**Affiliations:** ^1^Clinical Psychology and Neuropsychology, University of Konstanz, Konstanz, Germany; ^2^vivo international, Konstanz, Germany

**Keywords:** aggression, sensitive periods, appetitive aggression, demobilization, combatants, offender, adolescent, pubertal development

## Abstract

Violent behavior can be intrinsically rewarding; especially combatants fighting in current civil wars present with elevated traits of appetitive aggression. The majority of these fighters were recruited as children or adolescents. In the present study, we test whether there is a developmental period where combatants are sensitive for developing a robust trait of appetitive aggression. We investigated 95 combatants in their demobilization process that were recruited at different ages in the Kivu regions of the eastern Democratic Republic of Congo. Using random forest with conditional inference trees, we identified recruitment at the ages from 16 and 17 years as being predictive of the level of appetitive aggression; the number of lifetime, perpetrated acts was the most important predictor. We conclude that high levels of appetitive aggression develop in ex-combatants, especially in those recruited during their middle to late teenage, which is a developmental period marked by a natural inclination to exercise physical force. Consequently, ex-combatants may remain vulnerable for aggressive behavior patterns and re-recruitment unless they are provided alternative strategies for dealing with their aggression.

## 1 Introduction

Former combatants have been found to present with a proclivity toward aggressive behavior. This may be reactive, i.e., result from attempts to cope with potential threats. Recent investigations reported that former members of armed groups may describe violent acts as appealing and intrinsically rewarding; a phenomenon referred to as appetitive aggression (1). In most conditions, aggressive behavior seems to be driven by both the reactive and the appetitive form of aggression. In reactive aggression, such as when being threatened, violent behavior is thought to be an effort to reduce a state of negative emotional valence, while in appetitive aggression, violent behavior enhances arousal by increasing positive valence and may lead to further violence in an effort to maintain or increase arousal levels. It is plausible that the likelihood of perpetrating violent acts increases with the trait of appetitive aggression (2, 3). The rewarding properties of the violent acts in turn intensify the trait of appetitive aggression and consolidate a self-reinforcing linkage between the evocation of violent cues (in real or imaginary) and the lust in it. From a clinical point of view, the latter bears the potential for a “process” or “behavioral” addiction (4) – the compulsive approach of “mood-altering events” as seen for instance in sex or food addiction or in gambling (5, 6). Within these contexts, the fact of having been repeatedly traumatized additionally fuels the gateways to aggressive outbursts. Meanwhile, such changes in personality seems highly adaptive in hostile conditions like war and (para)military contexts rendering the development of alternative strategies to deal with one’s appetite for violence unnecessary.

In this study, we wanted to explore developmental periods sensitive for the emergence of a high trait in appetitive aggression. We assumed that the adolescent years [for a very recent review on changes in the adolescent brain, see Ref. (7)] might be of particular relevance in this respect given that the pubertal detachment process from the natal family occurs and with it opens a window to adapt to more hostile environments. Especially for male adolescents, confrontation with one’s own potency in the exertion of physical force plays an important role for the transition from boyhood to manhood. Furthermore, the desire to conquer one’s own inner fears and to identify with concepts like bravery, valor, heroism, and integrity are important in developing a congruent self-concept and achieving higher chances of reproductive success (8). A heightened sensitivity to salient cues, which in turn leads to more risky decisions and a tendency toward affective, incentive-based responsiveness may fuel these processes (9, 10). Substance use disorders (11) as well as anti-social behavior (12) typically emerge at this time.

To explore sensitive periods for the growth of trait aggression, we used data from adult combatants who had been recruited at various developmental stages and were active members of armed groups for varying periods in the Democratic Republic of Congo (DRC). The Kivu regions of eastern DRC have a long history of occupations by colonial and other external armed forces and intertribal fighting. Fueled by the Rwandan genocide, at least one million people fled into this region. In reaction to the creation of political and military organizations, Rwandan and Ugandan armies entered the DRC launching the First Congo War (1996–1998), immediately followed by the Second Congo War, also known as Africa’s Great War (1998–2002). This was formally brought to an end with the signing of the Lusaka Peace Accord in 1999. In 2000, a United Nations (UN) mission (MONUC, since 2010 MONUSCO; Mission de l’Organisation des Nations Unies pour la Stabilisation en République Démocratique du Congo) was deployed to the DRC. Despite the significant UN presence (13), fighting among various armed groups has continued in the eastern DRC (14). Children at various ages are forcibly recruited or join armed groups out of desperation (15). Their military life is then stipulated by daily exposure to frequently extreme forms of violence. Fellow soldiers become new family-like social systems. As there is an established link between appetitive aggression and perpetrated violent acts, we predict that the age of recruitment further potentiates appetitive aggression, with adolescents being especially likely to be imprinted by experiences of perpetration.

## 2 Materials and Methods

### 2.1 Participants

All Congolese ex-combatants who joined the MONUSCO Disarmament, Demobilization and Reintegration (DDR) program during the study period and who were older than 18 years (according to MONUSCO age test) were interviewed. In total, there were 95 participants ranging in age from 16 to 42 years. The majority of participants belonged to the Hutu (56%, *n* = 53), Nande (22%, *n* = 21), or Hunde (10%, *n* = 9) ethnic groups. They had served as combatants for between 1 month and 24 years (*M* = 57.2, *SD* = 55.5), the majority for different armed groups (two or more armed groups: 52%, *n* = 49). About two-thirds (65%, *n* = 62) reported that they had been forcibly recruited at least once, whereas 58% (*n* = 55) reported that they willingly joined an armed group (usually due to a lack of alternative financial resources). The median age of first recruitment was 17 years, range: 6–35 years). More than two-thirds (65%, *n* = 62) of the ex-combatants were recruited for the first time as children, i.e., before the age of 18 years. The variance in recruitment age was expected and thought to result in the required variance in trait of appetitive aggression.

### 2.2 Procedure

Interviews were conducted individually at a secluded place in the MONUSCO demobilization camp as part of the respective DDR program. Interviews lasted between 1.5 and 2.5 h. Participants gave their informed consent in writing; if illiterate, we asked for fingerprints. All of the subjects who were approached agreed to participate despite the fact that no reimbursement had been offered. All ex-combatants who arrived at the camp during the following time periods were interviewed and included in the analysis: February 2nd to 11th, February 27th to March 13th, and March 26th to April 5th, 2013. (It should be noted that life in the “bush” is extremely tough even during periods without combat, when there is frequently insufficient nutrition, very limited access to medical treatment, no possibility to lead a family life etc. The ex-combatants frequently want their voices to be heard and are ready to report to the outside world even if they see no immediate personal benefit. This explains the very high acceptance rate.) The ethical commission of the University of Konstanz, the board of the NGO vivo and MONUSCO approved the study. The questionnaires used in the study were translated into Kiswahili and back by independent groups of translators from Goma. The interviews were conducted by a group of local interviewers (one psychologist, four psychology students, and one translator). These interviewers were trained during an intensive ten-day session in the basic theoretical concepts underlying the research and in sensitive and empathic interviewing techniques. The interviewers received two follow-up training (3 and 7 days). Throughout the data collection periods, interviewers were closely supervised by clinical experts and received extensive feedback. All of the diagnostic instruments described in the following section were administered in the form of structured interviews.

### 2.3 Measures

Sociodemographic information included age, ethnicity, educational background, and details regarding the participant’s military career (group, year joined/left, and voluntary/forced recruitment).

#### 2.3.1 Exposure to Violence

A 31-item event checklist adapted from previous studies with ex-combatants in the Kivu regions [e.g., Ref. (15, 16)] was administered to assess lifetime exposure to different types of potentially traumatic events (experienced and witnessed) and perpetrated violent acts (war and non-war related). The total number of types of witnessed (possible range: 0–10) and experienced (possible range: 0–12) traumatic events and the number of types of perpetrated violent acts (possible range: 0–9) was calculated. Reliability measures showed that the applied event checklist had high inter-rater reliability (Cohen’s κ = 0.89).

#### 2.3.2 PTSD

Participants’ diagnostic status and PTSD symptom severity were assessed using the PTSD Symptom Scale-Interview [PSS-I; Foa and Tolin (17)]. The PSS-I is based on the 17 DSM-IV (18) symptom criteria for PTSD and measures symptom intensity during the previous month. PTSD severity was calculated by totaling symptom scores (scores range from 0 to 51). Internal consistency and inter-rater reliability revealed excellent values (Cronbach’s α = 0.89; *intraclass correlation coefficient*, *ICC* = 0.98).

#### 2.3.3 Appetitive Aggression

Appetitive aggression was assessed using the Appetitive Aggression Scale [AAS, Weierstall and Elbert (19)]. The AAS consists of 15 items, which are rated by responses on a five-point scale ranging from 0 (I totally disagree) to 4 (I totally agree). The items solicit information about participants’ perception of violence (e.g., “Is it exciting for you, if you make an opponent really suffer?”; “Once fighting has started, do you get carried away by the violence?”). The AAS has been successfully implemented (16, 20) and validated (19) in comparable East African samples. The AAS score is calculated by adding the scores of the 15 items (possible scores range from 0 to 60). Psychometric property measures indicated excellent internal consistency (Cronbach’s α = 0.91) and high inter-rater reliability (*ICC* = 0.96) in the present study.

#### 2.3.4 Analysis

We used random forest (21) embedded in a conditional inference framework [hereafter “conditional inference random forest” or RF-CI; Hothorn et al. (25)]. Unlike the classical random forest, the RF-CI does not display a bias toward predictors with many categories in the variable selection process (23). Following the principles of ensemble methods, a certain number of trees (ntree) are aggregated to compose the random forest. Each tree is built using binary splits of the previously subsampled data [subsampling rate = 63.2% (24)]. The splitting variable is chosen according to the strength of the association between the covariates and the outcome (24, 25) from a set of randomly preselected predictors [*p*, mtry, *p*/3; Grömping (26)]. Next, the importance of each predictor variable is ranked based on the ensemble of trees [conditional variable importance, *cvi*; Strobl et al. (23)]. The *goodness of fit* can be assessed using the out-of-bag data (OOB). The results are used to calculate a Pseudo-*R^2^* from the mean squared error (MSE) and the total sum of squares [SST; OOB-*R^2^* = 1-MSE/SST; Grömping (26)].

To explore the relative impact recruitment had at a specific age, we re-coded the assessed variable of the participants’ periods of military recruitment into dichotomous variables, referring to being in an armed group or not (i.e., recruitment of 6 years of age? yes/no, recruitment at 7 years of age? yes/no, and recruitment of 25 years of age? yes/no). For those younger than 25, we predicted “not recruited” and controlled with a variable coding the year(s) until/after 25. We also controlled for lifetime perpetrated violent acts. Any periods sensitive for developing appetitive aggression would result from the predictor rankings specified by the variable importance (*cvi*).

The random forest analysis was conducted using R (version 2.15.0). The implementation we used was cforest (22) from the R package party (27) with unbiased variable selection (22). Details, including code and results for the four RF-CI models, can be accessed from the Supplementary Material.

## 3 Results

The exposure to violence was high: means for the number of different types of traumatic events were *M* = 6.38, *SD* = 1.32, range: 0–8 for witnessed and *M* = 5.66, *SD* = 1.85, range: 1–10 for experienced stressors. Participants reported having perpetrated *M* = 4.44, *SD* = 1.80, range: 0–9 violent acts.

**Figure 1:**
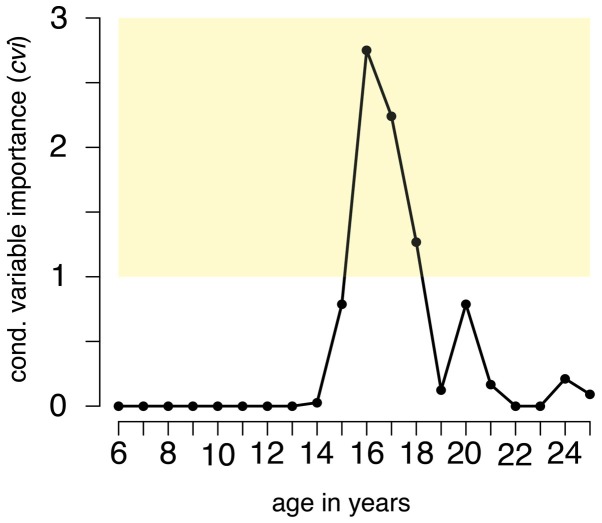
**Sensitive periods for developing a robust trait of appetitive aggression**.

RF-CI revealed that (para)military recruitment at the age of 16 years (*cvi* = 2.75), 17 years (*cvi* = 2.24), and 18 years (*cvi* = 1.27) does predict the level of appetitive aggression, with lifetime perpetrated acts being the most important predictor (*cvi* = 66.46). Notably, the (para)military recruitment at the ages of 6–14 years and above 20 years did not reach *cvi*s larger than 0.2 and are thereby negligible (see Figure 1). The explanation of variance from the OBB data is 31%. In a slightly varied model excluding lifetime perpetrated acts, 10% are explained by recruitment-related age-variables; being in an armed group at the age of 16 and 17 years remain the most important predictors and can, therefore, be considered as the most critical sensitive periods in the development of a robust trait of appetitive aggression. More details can be found in the Supplementary Material.

## 4 Discussion

In this study, we explored the periods sensitive for the development of appetitive aggression. Using RF-CI, we analyzed data from Congolese ex-combatants who had perpetrated various and frequently extreme forms of violence during their time as members of armed groups. In addition to the number of lifetime perpetrated violent acts, being recruited during late puberty, particularly at the ages of 16 and 17, turned out to be critical for the development of an appealing perception of violent cues; about 10% of variance is explained by these sensitive periods in our sample.

Currently, the number of child soldiers is estimated at 300,000 (UNICEF), and thereby the number of former child soldiers who are now adults is likely to be considerably higher. As implicated by our results, not only is their level of appetitive aggression expected to be elevated but they are also at a greater risk of violent behavior resulting in higher rates of familial violence, rape, and criminality as well as lower thresholds in rejoining an armed group (28). In fact, former combatants are a major source of destabilization in post-conflict regions (29). In order to rebuild a stable and peaceful society, it is important to provide interventions that also address the heightened attraction to aggression. Yet, the development of appropriate evidence-based treatment modules has only been recently attempted using an adapted version of Narrative Exposure Therapy (30–33, Köbach et al., submitted). Developing alternative strategies to deal with one’s craving for violence bears potential for further studies.

Moreover, this finding sheds light on the minimum age requirements in military service. Commonly, 18 years of age is the legal minimum age of (voluntary) recruitment. However, in the United States, Germany, the Netherlands, or Canada, young men are accepted by the age of 17 years and in the Great Britain, even a year earlier. In Great Britain, these young men can also be deployed in “war-fighting-situations” [Walker (34)]. Remarkably, 17% of British military personnel does have a criminal record later on (35)!

The result is in line with findings on the age of onset of anti-social personality disorder (12) as well as the age of onset of substance use disorders (11, 36), which, as with appetitive aggression, are driven by the shaping of neural reward circuits (1). With regard to findings in neuroscience, it seems that neural reward circuitry developed during adolescence (37) is more sensitive to cues associated with violence and thus may contribute to the emergence of a stable trait of appetitive aggression (38) recently introduced the so-called *positive affective neuroendocrinology* (PANE) approach integrating findings from neuroscience and endocrinology (mainly testosterone) research in terms of reward and behavioral dysregulation; indicating that these mechanisms are opening the window for sustainingly higher traits in appetitive aggression for young men who grow up in regions of civil war. However, these developmental dynamics of the neural and endocrinological correlates interacting with environmental inputs leading to a *love for battle* (39) remains to be explored.

Finally, it is important to note that the period of recruitment is only a rough measure of the point in time in which the violent acts had been executed. More precise measures may produce higher *cvi*s relative to other predictors and better fits to the OBB data.

## Author Contributions

AK supervised the data assessment, conducted the data analysis, and drafted the article. TE reviewed and revised the draft.

## Conflict of Interest Statement

The authors declare that the research was conducted in the absence of any commercial or financial relationships that could be construed as a potential conflict of interest.

## Supplementary Material

The Supplementary Material for this article can be found online at http://journal.frontiersin.org/article/10.3389/fpsyt.2015.00144

## Funding

vivo international, Deutsche Forschungsgemeinschaft (DFG), and European Research Council (ERC).
