# Supplementary material for: Sensitive Periods for Developing a Robust Trait of Appetitive Aggression
Source: Front Psychiatry. 2015 Oct 13;6:144. doi: 10.3389/fpsyt.2015.00144 (PMC4602097; doi:10.3389/fpsyt.2015.00144)
Supplement: Supplementary file 1 [file Presentation_1.ZIP › SOM.R.html]

Sensitive Periods in the Development of Appetitive Aggression


# Sensitive Periods in the Development of Appetitive Aggression

Content

1. Random forest regression on the level of appetitive aggression using DDR data (predictors: el\_p, age25, rec6-25)
2. Random forest regression on the level of appetitive aggression using DDR data (predictors: age25, rec6-25)

> Predictor variables:
>
> el\_p: number of lifetime perpetrated acts
>
> age25: number of years to/after being 25 years
>
> rec6: having been recruited at the age of 6 years
>
> rec7: having been recruited at the age of 7 years
>
> rec8: having been recruited at the age of 8 years
>
> rec9: having been recruited at the age of 9 years
>
> and so on until
>
> rec25: having been recruited at the age of 25 years

```
library(party)

set.seed(433)

forest.ddr <- cforest(as.numeric(aas_ss) ~ age25 + el_p + rec6 + rec7 + rec8 + 
    rec9 + rec10 + rec11 + rec12 + rec13 + rec14 + rec15 + rec16 + rec17 + rec18 + 
    rec19 + rec20 + rec21 + rec22 + rec23 + rec24 + rec25, data = ddr.data, 
    controls = cforest_unbiased(mtry = 7, ntree = 2000))

# Compute conditional variable importance

vic.ddr <- varimp(forest.ddr, conditional = TRUE)

write.table(vic.ddr)
```

```
"x"
"age25" 1.19187731749062
"el_p" 66.4644529947494
"rec6" 0
"rec7" 0
"rec8" 0
"rec9" 0
"rec10" -0.00775490196078431
"rec11" -0.00420995089493795
"rec12" -0.0352652209382313
"rec13" -0.137459718450389
"rec14" 0.0268928411981515
"rec15" 0.788146768441523
"rec16" 2.75091918577093
"rec17" 2.2405692323909
"rec18" 1.26771679617125
"rec19" 0.123797661204291
"rec20" 0.788358716835347
"rec21" 0.166266182938834
"rec22" -0.222997346909657
"rec23" -0.085917144999663
"rec24" -0.211904698798706
"rec25" 0.0911570964972486
```

```
plot(vic.ddr, frame = FALSE)
```

```
# Compute pseudo-R^2 from the out-of-bag-data

pred.ddr <- predict(forest.ddr, OOB = TRUE)

MSE.ddr <- mean((ddr.data$aas_ss - predict(forest.ddr))^2)
SST.ddr <- mean((ddr.data$aas_ss - mean(ddr.data$aas_ss))^2)
R_Sq.ddr <- (1 - (MSE.ddr/SST.ddr))
R_Sq.ddr
```

```
[1] 0.3127
```

```

```

```
library(party)

set.seed(433)

forest.ddr.1 <- cforest(as.numeric(aas_ss) ~ age25 + rec6 + rec7 + rec8 + rec9 + 
    rec10 + rec11 + rec12 + rec13 + rec14 + rec15 + rec16 + rec17 + rec18 + 
    rec19 + rec20 + rec21 + rec22 + rec23 + rec24 + rec25, data = ddr.data, 
    controls = cforest_unbiased(mtry = 7, ntree = 2000))

# Compute conditional variable importance

vic.ddr.1 <- varimp(forest.ddr.1, conditional = TRUE)

write.table(vic.ddr.1)
```

```
"x"
"age25" 2.03316123681267
"rec6" 0
"rec7" 0
"rec8" 0
"rec9" 0
"rec10" 0
"rec11" -0.00179621848739495
"rec12" -0.00477280338133178
"rec13" -0.304829079177737
"rec14" -0.584499636693862
"rec15" 1.77702971912987
"rec16" 4.1878017233877
"rec17" 2.42554002061973
"rec18" -0.0978702753406047
"rec19" 0.357635519661113
"rec20" 1.54442995007463
"rec21" 0.938771040106987
"rec22" -0.37500050857756
"rec23" -0.261369025349363
"rec24" -0.376414565595193
"rec25" -0.285303725787906
```

```
plot(vic.ddr.1, frame = FALSE)
```

```
# Compute pseudo-R^2 from the out-of-bag-data

pred.ddr.1 <- predict(forest.ddr.1, OOB = TRUE)

MSE.ddr.1 <- mean((ddr.data$aas_ss - predict(forest.ddr.1))^2)
SST.ddr.1 <- mean((ddr.data$aas_ss - mean(ddr.data$aas_ss))^2)
R_Sq.ddr.1 <- (1 - (MSE.ddr.1/SST.ddr.1))
R_Sq.ddr.1
```

```
[1] 0.09986
```

```

```
